# Two siblings with galactose mutarotase deficiency: Clinical differences

**DOI:** 10.1002/jmd2.12263

**Published:** 2021-11-29

**Authors:** Havva Yazici, Ebru Canda, Yasemin Atik Altınok, Sema Kalkan Ucar, Mahmut Coker

**Affiliations:** ^1^ Department of Pediatric Metabolism and Nutrition Ege University Faculty of Medicine Izmir Turkey

**Keywords:** cataract, galactose, galactose mutarotase, GALM, Leloir pathway

## Abstract

Galactose mutarotase (GALM) deficiency is an inherited metabolic disease caused by the deficiency of the first enzyme in the Leloir pathway. GALM deficiency was first reported in 2018. To date, eight cases have been reported. We are presenting two siblings with GALM deficiency; one patient presented with cataracts and her brother was asymptomatic. We evaluated the first case due to a cataract at 3 months old. She had elevated galactose and galactose‐1‐phosphate and normal galactose‐1‐phosphate uridylyltransferase (GALT) activity. Genetic analysis and other laboratory and clinical findings excluded galactokinase‐1 (GALK1) and UDP‐galactose 4′‐epimerase (GALE) deficiencies. She had a homozygous mutation p. Gly277Arg (c.829G>A) in the GALM (NM_138801) gene. She was 3 years old at the last visit, and her physical examination was normal, except for cataracts. The same mutation was found to be homozygous in the patient's asymptomatic sibling during family screening. He had normal blood galactose and galactose‐1‐phosphate. This study highlights the importance of evaluating the whole galactose metabolism in terms of GALM deficiency in patients with cataracts.

## 1 INTRODUCTION

Galactosemia is a metabolic condition caused by the lack of one of the four enzymes in the Leloir pathway. These four enzymes are galactose mutarotase (GALM), galactokinase (GALK1), galactose‐1‐phosphate uridyltransferase (GALT) and galactose 4‐epimerase (GALE), and their deficiencies cause the diseases galactosemia type‐4, type‐2, type‐1 and type‐3, respectively.^1^, ^2^

GALM catalyses the epimerisation between β‐d‐galactose and α‐d‐galactose in the first step of the pathway. The GALM gene is located on chromosome 2p22.1 and contains nine exons.^1^, ^3^, ^4^, ^5^, ^6^ Although galactosemia types‐1, 2, and 3 are well known, type‐4 (MIM: 137030) was first reported in 2018. They encountered eight patients with unexplained and persistent galactosemia. The estimated prevalence of type‐4 galactosemia is 1 in 228 411, but this varies widely between ethnic groups.^3^ The clinical presentations of reported patients are very diverse. They range from asymptomatic cases to congenital cataracts and transient cholestasis. All patients reported by Wada et al. followed a galactose‐restricted diet, but two patients discontinued the diet.^1^

Iwasawa et al. reported on the prevalence of *GALM* mutations that cause galactosemia by evaluating the Exome Aggregation Consortium Database (ExAC). They noted that GALM deficiency exists in many countries and observed unidentified pathogenic variants.^3^ Genetic diagnosis is required for diagnosing GALM deficiency because no biochemical diagnostic method has been determined.^1^

We are reporting on two siblings diagnosed with GALM deficiency. The index case had cataracts and elevation in galactose metabolites which could explain by the mutant GALM gene. On the other hand, her sibling had the same mutation but asymptomatic and normal galactose metabolites.

## 2 CASE REPORT

### 2.1 Patient 1

**FIGURE 1:**
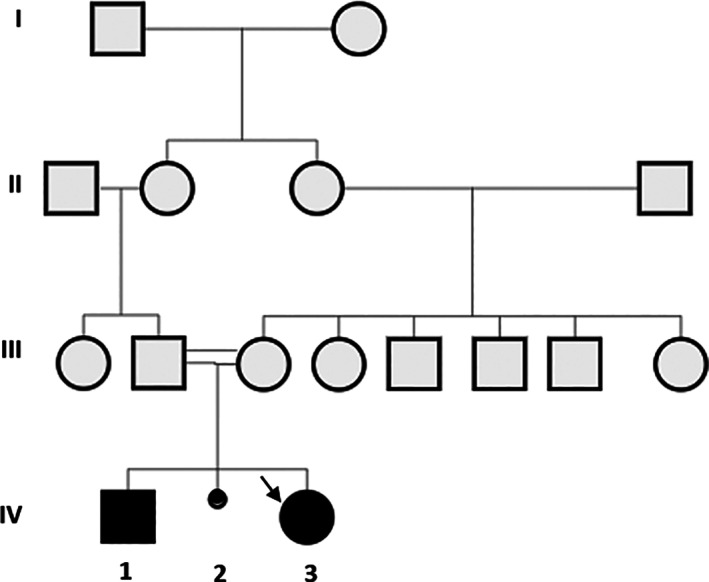
Pedigree of the patient family. 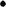
Spontaneous abortus. The arrow indicates the index case (Patient 1)

She was born to healthy consanguineous Turkish parents following an uneventful pregnancy and delivery. She was born at term with a birth weight of 2.920 g (−0.9 SD), a birth height of 48 cm (−0.66 SD) and a birth head circumference of 34 cm (−0.36 SD). She had an older brother. We detailed the family pedigree in Figure 1.

She was diagnosed with mild bilateral cataracts at her first ophthalmological examination at the age of 3 months. At her first physical examination at 3 months, body weight was in the 50th–75th percentile, height was 50th percentile and head circumference was 50th percentile. A bilateral cataract was detected, and examination of other systems was normal.

Her complete blood count was normal. We detected mildly elevated serum alanine transaminase and aspartate transaminase levels and normal bilirubin levels (Table 1). There was neither portosystemic shunt nor any abnormal findings on abdominal ultrasound.

**TABLE 1:** Biochemical features

	References range	Patient 1			Patient 2
		Three months (before diet)	One year (under diet)	Three years (under diet)	Six years (no diet)
Total galactose (mg/dL)	<10	**33.7**	1.7	4.4	3.1
Free galactose (mg/dL)	<5	**32.2**	0.8	1.3	1.9
Galactose‐1‐P (mg/dL)	<5	1,5	0.9	3.1	1.2
GALT (U/g Hb)	>3	4.3	10.1	11	8.5
ALT (U/L)	<34	**40**	19	22	25
AST (U/L)	<31	**52**	**41**	**44**	26

*Note*: Abnormal values were indicated in bold.

Blood total galactose and free galactose levels were elevated at 33.7 mg/dL (*N* < 10) and 32.2 mg/dL (*N* < 5), respectively (Table 1). The GALT activity and Gal‐1‐P level were normal at 3 months of age. An assay for GALK1 activity was not available, so we excluded GALK1 deficiency using genetic analysis. GALE deficiency was excluded by *GALE* gene analysis. Urinary reducing substances test, urine amino acid test and urine glucose test results were normal. Due to abnormal galactose metabolites, whole‐exome sequencing was carried out as part of a differential diagnosis and detected homozygous c.[829G>A];[(829G>A)], p.(Gly277Arg);(Gly277Arg) pathogenic variants in the GALM gene. We confirmed these variants through Sanger sequencing of the parents.

We started galactose restriction at the age of 106‐days, and she remains on a galactose‐restricted diet. The blood total galactose, free galactose and Gal‐1‐P levels were within the normal range (Table 1). Even though total galactose and free galactose, high before starting treatment, returned to normal after treatment, cataracts did not resolve. She had to have an operation due to cataracts at the age of 6 months. She showed normal growth and neuromotor development at the most recent follow‐up when she was 3 years old.

### 2.2 Patient 2

Patient 2 (IV‐1) was a 6‐year‐old boy born to healthy, consanguineous parents (Figure 1). He was born at term with average birth weight and no adverse perinatal events. He had normal growth and reached typical neuromotor milestones. He had no hepatomegaly. His ophthalmologic examination was regular except for hypermetropia. The other system examinations were all normal. Liver function tests and urine glucose analyses were all normal. Bilirubin levels were normal, and abdominal ultrasound did not reveal abnormal findings. We performed Sanger sequencing analysis of the *GALM* gene due to his sister being diagnosed with GALM deficiency. *GALM* gene analysis showed that he was homozygous for p.(c.829G>A)/p.Gly277Arg mutation. The blood galactose and Gal‐1‐P levels were normal under a non‐restricted diet at the time of the diagnosis. We did not have any information about the lactose content of his daily nutrition. We had no information about galactose metabolites during the neonatal period. We did not start a galactose‐free diet for GALM deficiency due to his normal clinical and laboratory findings. The patient continues to be followed‐up.

## 3 DISCUSSION

After the first clinical report presented in 2018 from Japan, no cases have been reported to date to the best of our knowledge. Our two cases show that clinical pictures can vary in GALM deficiency even with the same mutation in siblings. This finding is similar to what is reported in the literature.^1^ Clinicians should perform differential diagnoses of GALT, GALK1 and GALE according to the clinical picture and abnormal investigations with abnormal galactose metabolites. Usually, GALT, GALK1 and GALE tend to be kept in mind in the differential diagnoses of patients who have abnormal galactose metabolites. According to the clinical picture and laboratory findings, GALM deficiency should be ruled out in such patients. To date, genetic diagnosis is required to diagnose GALM deficiency since there is no biochemical diagnostic method available to us. Wada et al. reported five mutations in eight GALM deficiency cases to date.^1^ *GALM* gene analysis showed that both of the patients presented were homozygous for (c.829G>A)/(p.Gly277Arg). Iwasawa et al. reported the prevalence of *GALM* mutations that cause galactosemia by evaluating the ExAC, and they predicted that (c.829G>A)/(p.Gly277Arg) mutation was a pathogenic mutation.^3^ GALM deficiency exists in many countries, and there remain unidentified pathogenic variants.^1^, ^3^, ^4^

Wada et al. reported two patients with GALM deficiency who had congenital cataracts detected at 7 and 10 months (first ophthalmological examination). Our Patient 1 was diagnosed with a congenital cataract at her first ophthalmological examination at 3 months old. Even though we started a restricted galactose diet when the patient was 106 days old, the cataract did not resolve under dietary treatment. She underwent a bilateral cataract operation. One of Wada's patient's cataracts resolved after the start of a galactose‐restricted diet. Starting the treatment at an early age (44 days) may explain the resolution of a cataract. By including galactosemia in NBS programs, we can prevent cataract development with early diagnosis and treatment.

However, we know that GALM deficiency may present as persistent congenital cataracts while gastrointestinal symptoms or severe liver dysfunction are absent.^1^ The natural history of the disease is unknown. Wada et al. reported two patients with transient cholestasis and two patients with a spontaneously regressed portosystemic shunt which was a coincidental finding. We did not detect hepatomegaly in our patients, similar to what is reported in the literature. According to the literature, transient cholestasis is also an important finding in GALM deficiency.^1^ Our cases did not have transient cholestasis or portosystemic shunt. Also, Wada et al. reported that none of the eight patients presented with severe liver dysfunction.^1^ Our Patient 1 had persistent mildly elevated liver enzymes, while the second patient did not show symptoms of liver dysfunction. We do not know whether mildly elevated liver enzymes are related to GALM deficiency.

None of the patients with GALM deficiency had problems with growth or neurodevelopment. The existence of long‐term complications such as neurocognitive problems and premature ovarian failure was not yet known because no adult patients had been reported so far.^1^, ^2^, ^3^

All of the patients reported by Wada et al. had a galactose‐restricted diet before 2 months of age. Two of their patients discontinued a restricted galactose diet at 18 months, and the age of 11 years. The galactose and Gal‐1‐P levels were maintained in the normal range in one patient, even after stopping the restriction. However, the galactose level increased in the other patient, where the restriction was relaxed, whereas the Gal‐1‐P level was in the normal range.^1^ The mechanism of Gal‐1‐P fluctuations in patients with GALM deficiency is still unknown. The other unknown point is whether GALM has a role solely in the Leloir pathway or if it also has a role in the metabolism of other monosaccharides.^1^, ^4^

## 4 CONCLUSION

The pathophysiology, clinical findings, and treatment modality associated with the GALM deficiency are not clear. Physicians should consider this disorder in the presence of cataracts or temporary cholestasis, in line with the data from the literature. Cases have been reported with and without a galactose‐restricted diet. It is necessary to decide on the diet to be applied according to the patient. As the number of patients increases, we will have more information about the course of the disease and its clinical diversity.

## CONFLICT OF INTEREST

The authors declare no conflict of interest.

## ETHICS STATEMENT

The work described has been carried out in accordance with The Code of Ethics of the World Medical Association (Declaration of Helsinki, 2013). Informed consent was obtained from the patients' parents for being included in the study.

## Data Availability

The manuscript has no associated data.
